# Transfer hydrogenation catalysis in cells as a new approach to anticancer drug design

**DOI:** 10.1038/ncomms7582

**Published:** 2015-03-20

**Authors:** Joan J. Soldevila-Barreda, Isolda Romero-Canelón, Abraha Habtemariam, Peter J. Sadler

**Affiliations:** 1 Department of Chemistry, University of Warwick, Gibbet Hill Road, Coventry CV4 7AL, UK

## Abstract

Organometallic complexes are effective hydrogenation catalysts for organic reactions. For example, Noyori-type ruthenium complexes catalyse reduction of ketones by transfer of hydride from formate. Here we show that such catalytic reactions can be achieved in cancer cells, offering a new strategy for the design of safe metal-based anticancer drugs. The activity of ruthenium(II) sulfonamido ethyleneamine complexes towards human ovarian cancer cells is enhanced by up to 50 × in the presence of low non-toxic doses of formate. The extent of conversion of coenzyme NAD^+^ to NADH in cells is dependent on formate concentration. This novel reductive stress mechanism of cell death does not involve apoptosis or perturbation of mitochondrial membrane potentials. In contrast, iridium cyclopentadienyl catalysts cause cancer cell death by oxidative stress. Organometallic complexes therefore have an extraordinary ability to modulate the redox status of cancer cells.

## Abstract (web summary)

Organometallic complexes are effective hydrogenation catalysts for organic reactions. Here the authors report for the first time that transfer hydrogenation catalysis can take place inside the cell and could be used as a novel anticancer strategy.

## Graphical abstract

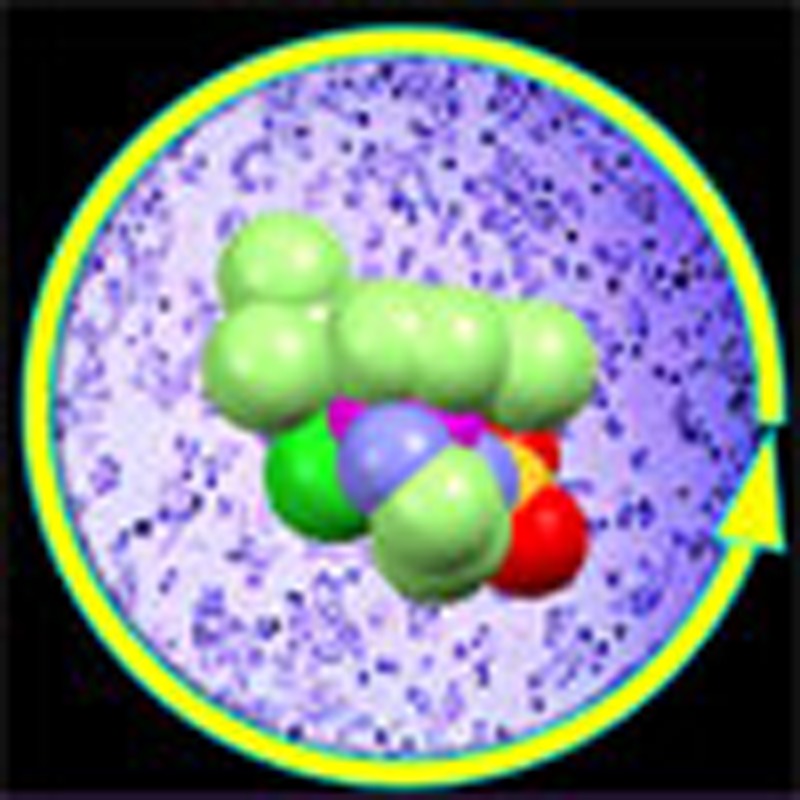

Organometallic complexes are well known as catalysts for organic chemical reactions, for example, olefin metathesis by Grubbs’ ruthenium carbene complexes^1^,^2^ and asymmetric hydrogenation of ketones by Noyori’s ruthenium arene complexes^3^,^4^. The prospect of using organometallic complexes as catalytic drugs is attractive since this might allow low safe non-toxic doses of transition metals to be administered, and furthermore introduce novel mechanisms of action that can overcome resistance to current widely used platinum anticancer drugs^5^,^6^,^7^. However, achieving catalytic activity in cells is challenging on account of the presence of many nucleophilic biomolecules, which might act as catalyst poisons. Here we show for the first time that Noyori-type ruthenium complexes can catalytically reduce coenzyme NAD^+^ in human ovarian cancer cells using non-toxic concentrations of formate as a hydride donor. Moreover, such catalysis greatly enhances the potency of the complexes and increases selectivity towards cancer cells *versus* normal cells. The mechanism of cancer cell death involves the generation of reductive stress, in contrast to organometallic iridium cyclopentadienyl anticancer catalysts, which induce oxidative stress^8^. It is apparent that organometallic complexes have a unique ability to modulate the redox status of cells.

First we considered the choice of organometallic transfer hydrogenation catalysts for cell studies. Several Ru^II^, Rh^III^ and Ir^III^ complexes have previously been reported to catalyse the regioselective reduction of NAD^+^ to 1,4-NADH in water, using formate as a hydride source^9^,^10^,^11^. Stekhan and Fish *et al*. elucidated the mechanism of reduction of NAD^+^ using Cp*Rh^III^ bipyridine complexes (formate coordination, loss of CO_2_, hydride transfer to Rh^III^, then to NAD^+^ in a weak-association complex)^12^,^13^,^14^,^15^,^16^. Süss-Fink *et al*.^17^ reported the catalytic reduction of NAD^+^ using a series of Ru^II^, Rh^III^ and Ir^III^ phenanthroline catalysts. [(Cp*)Rh(phen)Cl]^+^ exhibits turnover frequencies (TOFs) of up to twice those of [(Cp*)Rh(bipy)Cl]^+^ in aqueous media^17^. Rh^III^ and Ru^II^ catalysts bearing dipyridyl amine ligands functionalized with maleimide are also active, but not as active as (Cp*)Rh(bipy)Cl]^+^ (ref. 18). In 2012, Hollmann *et al*.^19^ reported a tethered Rh^III^ complex containing an analogue of the Noyori ligand TsDPEN immobilized in a poly(ethylene) polymer. The catalytic activity of the heterogeneous catalyst was lower than that of other soluble Rh^III^ complexes.

Initially, we investigated the use of Rh^III^ sulfonamido ethyleneamine complexes for catalytic hydrogenation of NAD^+^ in cancer cells but found them to be less effective than the Ru^II^ analogues, which were therefore explored in more detailed studies. We showed previously that the efficiency of [(η^6^-arene)Ru(R-SO_2_-En)Cl] catalysts depends on both the nature of the arene and on the sulfonamide substituent (R)^20^. For cell work, we compared complexes **1**–**4** with *p*-cymene (*p*-cym) as the arene and the sulfonamide substituent R=methyl (MsEn, **1**), *p*-methylbenzene (TsEn, **2**), *p*-trifluoromethylbenzene (TfEn, **3**) and *p*-nitrobenzene (NbEn, **4**), with analogues **5**–**7** containing *o*-terphenyl (*o*-terp) as the arene and R=methyl (MsEn, **5**), *p*-methylbenzene (TsEn, **6**), *p*-trifluoromethylbenzene (TfEn, **7**), Fig. 1. The *o*-terp arene was chosen on account of its increased hydrophobicity (Supplementary Table 1) to enhance uptake into cells. The Ru^II^-ethylenediamine complex **8** was studied for comparison since this compound has poor catalytic activity, but good anticancer activity *in vitro* and *in vivo*^21^.

## Results

Regioselective catalytic reduction of NAD^+^(2 mol equiv) to 1,4-NADH was observed in MeOH*-d*_*4*_/D_2_O (2:9 v/v) and D_2_O alone using complexes **1**–**4** and formate as the hydride source (25 mol equiv) by ^1^H nuclear magnetic resonance (NMR) at 310 K, pH* 7.2±0.1. The TOFs for the reactions (Table 1, determined as described in the Methods section) showed a general trend in which the more electron-withdrawing sulfonamides gave higher catalytic activity: NbEn>TfEn>TsEn>MsEn. Such a trend has been previously reported^20^,^22^,^23^. The complexes studied in the present work show comparable TOFs (*ca*. 0.2–7 h^−1^) for the reduction of NAD^+^ to those reported for [(η^6^-arene)Ru(*N,N′*)Cl] complexes (*ca*. 0.006–10 h^−1^), although much lower than those obtained using Rh^III^ or Ir^III^ complexes^17^,^18^.

The catalytic reduction of NAD^+^ using complexes **5**–**7** under the same conditions was more rapid, being complete by the time the first ^1^H NMR spectrum was recorded. The reaction was therefore then carried out using only 6 mol equiv of NAD^+^, and the catalytic reactions were then complete within the first 10 min (**5**–**7**). Subsequently, formation of a brown precipitate was observed and, as a consequence, the TOF of these complexes could not be determined. However, it was apparent that the *o*-terp complexes were more active as catalysts than the *p*-cym complexes (for which the highest activity was observed for **4**, 20.5 min for completion).

The catalytic mechanism was expected to involve initial binding of formate to Ru followed by transfer of hydride and (irreversible) release of CO_2_. Evidence for this was provided by complex **5** for which a Ru-H ^1^H NMR peak was detected at −5.5 p.p.m. (Supplementary Fig. 1) as well as mass spectrometry (MS) peaks assignable to both the formate and hydride adducts (Supplementary Table 2).

**Figure 2:**
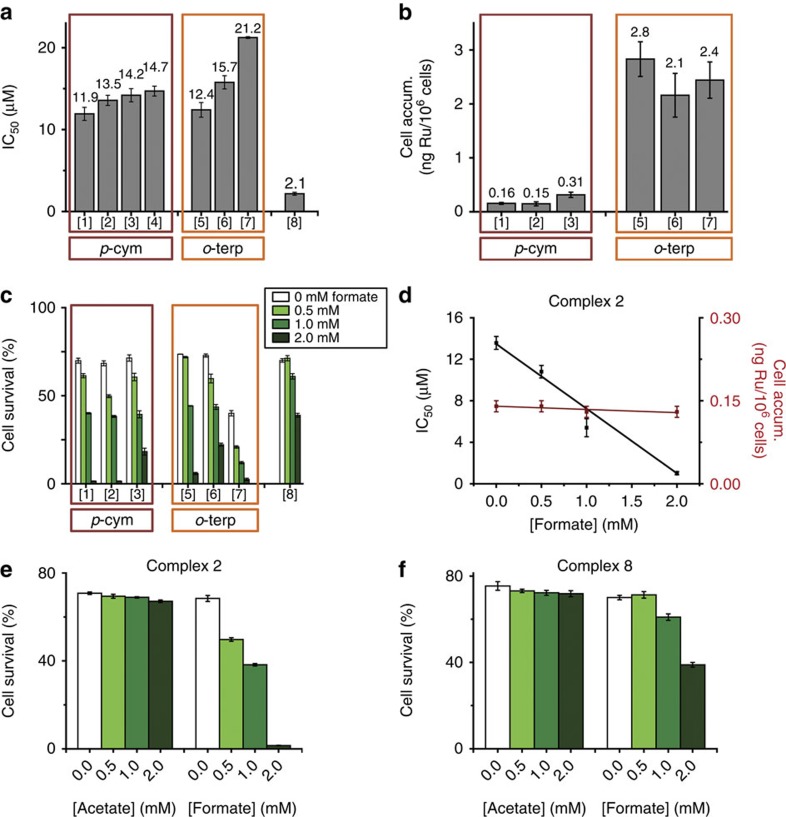
Antiproliferative activity in A2780 human ovarian cancer cells. (**a**) IC_50_ values for complexes **1**–**8**. (**b**) Cellular accumulation of Ru from *p*-cym complexes **1**–**3** and their *o*-terp analogues **5**–**7** after 24 h. (**c**) Percent cell survival when equipotent concentrations of complexes **1**–**3** and **5**–**8** (1/3 × IC_50_) were co-administered with different concentrations of sodium formate. (**d**) IC_50_ values for complex **2** when co-administered with various concentrations of sodium formate and the cellular accumulation of Ru under similar conditions. (**e**) and (**f**) Comparison of cell survival (%) for complexes **2** and **8** when co-administered with various concentrations of sodium acetate or sodium formate. All experiments included 24 h of drug exposure. All the experiments were performed as duplicates of triplicates in independent experiments and the error bars were calculated as the s.d. from the mean.

We then investigated the antiproliferative activity of complexes **1**–**7** in A2780 human ovarian cancer cells, and compared them with both complex **8** (RM175) and the clinically approved drug cisplatin. Complexes **1**–**4** were moderately active with half-maximal inhibitory concentration (IC_50_) values ranging from 11.9 to 14.7 μM (Fig. 2; Supplementary Table 3), some 6–12 × less potent than complex **8** (2.2 μM) and cisplatin (1.2 μM). The *o*-terp complexes **5**–**7** were slightly less active, with IC_50_ values in the range 12.4–21.2 μM. The potency order in both series is MsEn>TsEn>TfEn, the reverse of the order seen for catalytic activity above. However, no correlation would be expected since these antiproliferative experiments were not done under catalytic conditions.

The most notable difference in the properties of the ethylenediamine complex **8** and the sulfonamido ethyleneamine complexes **1**–**7** is the ability of the sulfonamide substituent to stabilize an adjacent deprotonated Ru-bound N and in turn 16e^−^ intermediates in the catalytic cycle. We thought that this change in electronic distribution would affect DNA binding, which is thought to play a major role in the activity of complex **8**. Complex **8** binds strongly to guanine bases in DNA after initial aquation^24^,^25^,^26^,^27^,^28^,^29^,^30^,^31^.

^1^H NMR studies showed that aquation of complexes **1**–**7** was complete in <5 min at 298 K, and therefore faster and more favourable than for **8**. As might be expected from the increased charge density on Ru in the sulfonamide complexes, the pK_a_ values of the aqua adducts of complexes **1**–**7** were higher (by about two units, range 9.5–9.9, Table 1; Supplementary Fig. 2) than for aquated **8** (pK_a_ 7.7). The sulfonamido complexes would therefore exist mainly as aqua adducts over the pH range used in the present experiments (close to physiological, pH 7).

Perhaps surprisingly, the model base 9-ethylguanine had a similar affinity for complexes **1**–**4** as complex **8** (as determined by ^1^H NMR, equilibrium constants 60–105 mM^−1^ compared with 60 mM^−1^ for **8**, Supplementary Table 4), and binding (to N7, Supplementary Fig. 3) was rapid (pH* 7.2, 310 K). The adducts were also characterized by electrospray ionization–MS (Supplementary Table 5). No binding to 9-methyladenine was detected. However, no binding of complex **2** (as a representative of the series) to calf thymus DNA was detected by inductively coupled plasma-mass spectrometry (ICP–MS) measurements of bound Ru even after a 27-h incubation at 310 K (mol ratio 1:3 complex:DNA base pairs, 2 mM cacodylate buffer pH 7.4, 2 mM NaCl). The absence of strong binding to calf thymus DNA was also apparent from circular and linear dichroism and melting temperature studies (Supplementary Table 6; Supplementary Figs 4 and 5). This weak binding is probably a consequence of unfavourable steric and electronic interactions between the DNA double helix and the sulfonamido side chain in comparison with the unsubstituted ethylenediamine analogue **8**. Hence, we can conclude that DNA is not likely to be a target for the sulfonamide complexes.

**Figure 3:**
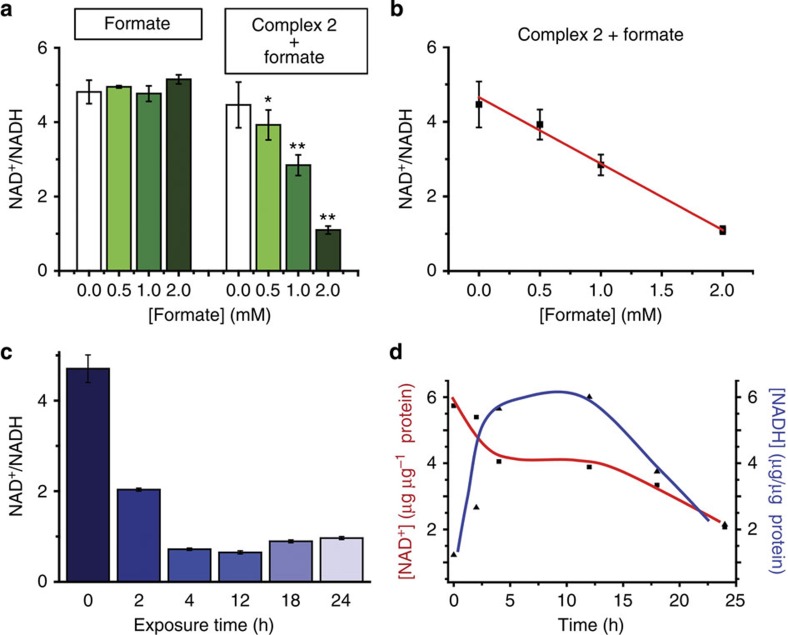
Perturbation of the NAD^+^/NADH ratio in A2780 cells exposed to 1/3 × IC_50_ of complex 2 and sodium formate. (**a**) Variation in the concentration of formate (0, 0.5, 1 and 2 mM) in the absence (left) and presence (right) of complex **2**, 24 h exposure. (**b**) Graph showing the linear correlation between the concentration of sodium formate and the NAD^+^/NADH ratio. (**c**) Effect of exposure time (0, 2, 4, 12, 18 and 24 h) on the NAD^+^/NADH ratio. (**d**) Variation in the concentration of NAD^+^ and NADH with time. All the experiments were performed as duplicates of triplicates in independent experiments and the error bars were calculated as the s.d. from the mean. An independent two-sample *t*-test with unequal variances, Welch’s test, was used to define the statistical difference between the values obtained for the NAD^+^/NADH ratio experiment. *P<0.05, **P<0.01.

Next, we investigated the antiproliferative activity of the complexes towards A2780 human ovarian cancer cells in the presence of formate, conditions under which catalytic intracellular conversion of NAD^+^ to NADH might occur. First we showed that formate itself at concentrations from 0 to 2 mM is non-toxic to the cells (Fig. 3). Then A2780 cells were co-incubated with equipotent concentrations of complexes **1**–**8** (1/3 × IC_50_) and three different concentrations of sodium formate (0.5, 1 and 2 mM). The antiproliferative activity of complexes **1**–**7** was enhanced by co-administration with formate (Supplementary Tables 7 and 8; Fig. 2). The degree of enhancement of activity was directly proportional to the formate concentration (Fig. 2), consistent with a direct contribution to the activity from catalytic transfer hydrogenation. The antiproliferative activity of complex **8** was also enhanced by the co-administration of formate, but to a much lower extent (Fig. 2). The largest decrease in cell survival, of *ca*. 50 × , was observed for complexes [(*p*-cym)Ru(MsEn)Cl] (**1**) and [(*p*-cym)Ru(TsEn)Cl] (**2**), dropping from *ca*. 69% to 1% when the concentration of formate was increased from 0 to 2 mM (Fig. 2). Complexes containing *o*-terp also showed a significant enhancement of antiproliferative activity, with complex **7** giving the greatest decrease in cell survival (*ca*. 16-fold). In contrast, the presence of formate only decreased cell survival induced by complex **8** (RM175) from *ca*. 70 to 39%.

Next, we investigated whether the formate-induced increase in activity was accompanied by intracellular conversion of NAD^+^ to NADH using the most active complex **2**. Cellular accumulation of ruthenium in A2780 cells exposed to complex **2** (4.5 μM) showed no significant dependence on the amount of formate added (Supplementary Table 8; Fig. 2), consistent with a direct relationship between the potentiation of activity and the ability of the formate to act as a hydride source. Furthermore, the cellular distribution of Ru from complex **2** (4.5 μM) was very similar in the presence and absence of formate (2 mM), being, in both cases, greatest in the cytosolic fraction (51%)>membrane and organelle fraction (38%)>>nuclear fraction (9%). There was little Ru in the cytoskeletal fraction (Supplementary Table 9).

The IC_50_ of complex **2** in A2780 cells decreased from 13.6±0.6 (no formate) to 1.0±0.2 μM in the presence of 2 mM formate (Fig. 2), notably achieving the potency of the clinical drug cisplatin, but having a different mechanism of action. Importantly, co-incubation with formate seems to increase the selectivity factor of complex **2** (from 3.6±0.2 to 5±1, Supplementary Table 10). Selectivity factors were calculated as the ratio between the antiproliferative activity towards normal cells (MRC5 fibroblasts) and the activity in A2780 ovarian cancer cells.

Cells, and living organisms in general, maintain a tight balance for their redox system by controlling the levels of oxidants and antioxidants. In particular, cancer cells are under constant oxidative stress, and high levels of reactive oxygen species such as OH, O_2_^−^ and H_2_O_2_ are common as a result of disturbed mitochondrial function^32^,^33^. As a consequence, neoplastic tissues are especially sensitive to further changes in the redox balance, for example, those caused by changes in the NAD^+^/NADH ratio. In contrast, non-cancerous cells have normal-functioning mitochondria that allow them to adjust to changes in their redox balance. This difference provides a strategy for conferring selectivity on anticancer agents that attack cancer cell metabolism^34^,^35^,^36^.

The specific role of formate was confirmed by comparison with acetate. The activity of neither complex **2** nor **8** towards human ovarian cancer cells was affected significantly by acetate under similar conditions (Fig. 2; Supplementary Table 11). Acetate, unlike formate, cannot act as a source of hydride for catalytic hydrogenation reactions.

We then investigated whether complex **2** in combination with formate could convert NAD^+^ to NADH in A2780 human ovarian cancer cells. In the first experiment, A2780 cells were incubatedfor 24 h with complex **2** and three different concentrations of sodium formate (0.5, 1 and 2 mM). Neither the complex nor formate alone had a significant effect on the intracellular NAD^+^/NADH ratio (Fig. 3). However, the NAD^+^/NADH ratio decreased significantly after co-administration of **2** and increasing amounts of formate (from 4.5 to 1.1 with 2 mM formate, Fig. 3).

An apparent TOF (TOF_ap_) of 0.19±0.01 h^−1^ was determined for the conversion of NAD^+^ to NADH in A2780 cancer cells treated with 4.5 μM complex **2** and 2 mM sodium formate for various times (0, 2, 4, 12, 18 and 24 h). However, the interpretation of this value is not straightforward since many other processes in cells may influence the concentrations of NAD^+^ and NADH. The NAD^+^/NADH ratio decreased rapidly for the first 4 h, from 4.78 (*t*=0 h, untreated) to 0.83 (*t*=4 h), followed by a 4-h period in which the decrease was less pronounced, reaching a final ratio of 0.74 after 12 h (Fig. 3). After 12 h, a slight recovery was observed, with the ratio reaching 0.98 after 24 h. This might be due to gradual poisoning of the catalyst (reaction with other biomolecules) or degradation.

**Figure 4:**
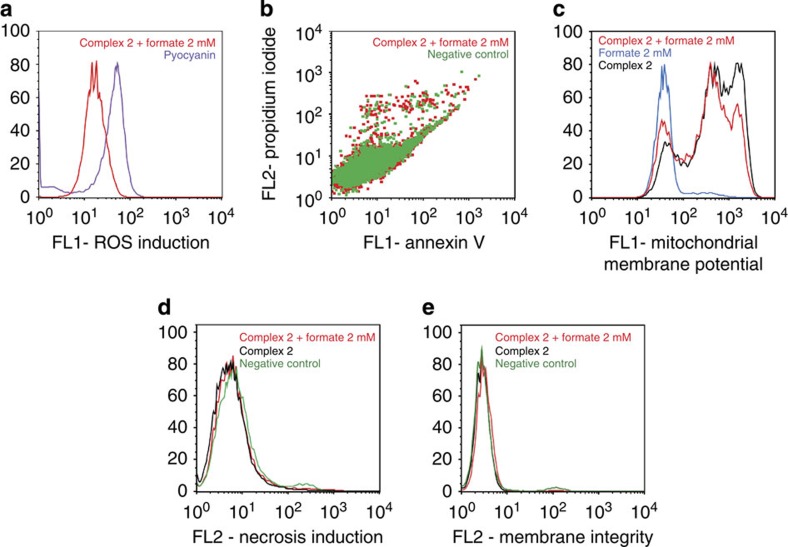
Mechanism of action studies on A2780 ovarian cancer cells. All experiments used 24 h of drug exposure time, fixed drug concentrations (13.6 μM complex **2**, 2 mM sodium formate) and no recovery time. (**a**) Induction of ROS in cells, pyocyanin was used as a positive control; (**b**) induction of apoptosis; (**c**) changes in the mitochondrial membrane potential; (**d**) induction of necrosis and (**e**) changes in cellular membrane integrity.

Flow cytometry analysis of A2780 cells exposed to complex **2** and 2 mM sodium formate showed that the levels of reactive oxygen species (ROS) in treated cells are comparable to those arising from treatment with *N*-acetyl-L-cysteine, a well-known reductant (Fig. 4a). The intracellular generation of ruthenium hydride species may lead not only to the reduction of NAD^+^ (and NADP^+^), but also to the reduction of other biomolecules such as ketones and imines^20^,^37^,^38^.

Induction of apoptosis in A2780 cells exposed to complex **2** and sodium formate was investigated using flow cytometry. Annexin V-fluorescein isothiocyanate (FITC) and propidium iodide (PI) dual-staining allowed the detection of four different populations: viable cells, non-viable cells and early-stage and late-stage apoptosis. Early-stage apoptosis is characterized by changes in the symmetry of the phospholipid membrane, and late stage by further disruption of the integrity of the cell membrane so it becomes permeable to PI. In the case of cells exposed to complex **2** and 2 mM of sodium formate, no population of cells in either of these two stages of apoptosis was detected after 24 h (Fig. 4b), as was the case after treatment with sodium formate alone.

We also used flow cytometry to investigate whether exposure of cells to complex **2** and sodium formate induced variations in the mitochondrial membrane potential. The induction of apoptosis is closely related to mitochondrial changes. In the intrinsic pathway of apoptosis activation, changes in the mitochondrial membrane potential allow release of cytochrome *c* into the cytosol, which promotes the subsequent activation of the caspase cascade^39^. No changes in the mitochondrial membrane potential were observed (Fig. 4c). This is consistent with a mechanism of action based on the perturbation of the redox state of cells, and the lack of apoptosis.

In the absence of apoptosis after 24 h of drug exposure, we investigated the induction of necrosis by flow cytometry. A2780 cells exposed to complex **2** in the presence and absence of 2 mM sodium formate tested negative for increased fluorescence of the 7-ADD stain in the FL2-red channel, indicating that, under these experimental conditions, there is no cellular necrosis either (Fig. 4d). Furthermore, the same technique was used to confirm cellular membrane integrity. In this case, cells exposed to complex **2** alone or in combination with 2 mM sodium formate showed low fluorescence in the FL2 channel reading for CytoPainter red, which reacts with exposed amines in compromised membranes (Fig. 4e). Other mechanisms that could be considered in future work include the induction of autophagy^40^ and pathogenic mitochondrial oxidation^41^, both of which have been linked to reductive stress, but it is also possible that the cell death observed here involves new pathways and cannot be simply mapped onto known mechanisms.

## Discussion

It is challenging to design metal complexes that might exhibit catalytic activity in living cells^5^,^6^,^7^. Catalysts for chemical transformations work efficiently under well-defined conditions, whereas cells contain a wide range of nucleophiles, as well as oxidants and reductants, which might readily poison the catalyst *via* substitution or redox reactions. Nevertheless, some success has been reported^42^,^43^,^44^,^45^. For example, manganese macrocycles such as M40403 can act as superoxide dismutase mimics and decompose superoxide into O_2_ and H_2_O_2_ in cells^46^,^47^. Iron porphyrin complexes can catalyse the reduction of azides to imines^48^, and copper peptide complexes can catalyse the degradation of RNA in hepatitis models^42^,^49^. Cobalt complexes, which cleave peptide bonds^50^,^51^,^52^, can decompose amyloids present in diseases such as Alzheimer’s or Parkinson’s^53^,^54^. In particular, organometallic compounds have shown success in the last few years. For example, Ru complexes such as [(η^6^-arene)Ru(azpy)I]^+^ (azpy=*N*,*N*-dimethylphenyl- or hydroxyphenyl-azopyridine) catalytically oxidize glutathione (GSH) to glutathione disulfide (GSSG), increasing the levels of ROS in cells^55^.

Meggers *et al*.^56^,^57^ have also shown that organoruthenium complexes such as [(Cp*)Ru(COD)Cl] (COD=cyclooctadiene) or the photoactivatable complex [(Cp*)Ru(η^6^-pyrene)]PF_6_ can catalyse the cleavage of allylcarbamates from protected imines. More recently, the catalytic cleavage of allylcarbamates in cells has been greatly improved by the use of complexes of the type [(Cp)Ru(QA-R)(η^3^-allyl)]PF_6_ (QA=2-quinolinecarboxylate; R=π-donating groups). The latter complexes can activate protected fluorophores or even protected anticancer drugs in cells^58^, an approach that could also provide reduction in toxicity for anticancer drugs. An interesting strategy for avoiding poisoning and increasing the stereoselectivity of organometallic catalysts is to incorporate them in the pocket of a host protein, as illustrated by Ward *et al*. using biotin-labelled Noyori-type Ir^III^ complexes and streptavidin as the protein host^59^.

In this work, we have shown for the first time that Noyori-type catalytic transfer hydrogenation can be achieved in living cells, using Ru^II^ arene complexes with a chelated sulfonylethylamine ligand and formate as the hydride donor to convert coenzyme NAD^+^ into NADH, and thereby modulate the NAD^+^/NADH redox couple.

Formate at non-toxic concentrations not only initiates the catalysis, but also enhances the anticancer potency of the complexes by a factor of up to *ca*. 14. Furthermore, this seems to be accompanied by an increase in selectivity, which for complex **2** rises from 3.6±0.1 to 5±1 in the presence of formate (*P*<0.01 in a Welch’s test). This introduces a new design concept for transition metal complexes, one which may allow therapeutic doses to be kept low and safe with minimal side effects. Moreover, the mechanism of cell death, reductive stress through conversion of coenzyme NAD^+^ to NADH, is unusual and different from that of cisplatin and most other anticancer agents. Hence, this new approach should be effective for treatment of cisplatin resistance, which has become a major clinical problem. Apoptosis is usually thought to be the main mechanism of cisplatin-induced cell death, but flow cytometry experiments on A2780 human ovarian cancer cells, together with the lack of changes in the mitochondrial membrane potential, ruled out the induction of apoptosis as the mechanism of cell death for the catalytic Ru complexes studied here.

**Figure 5:**
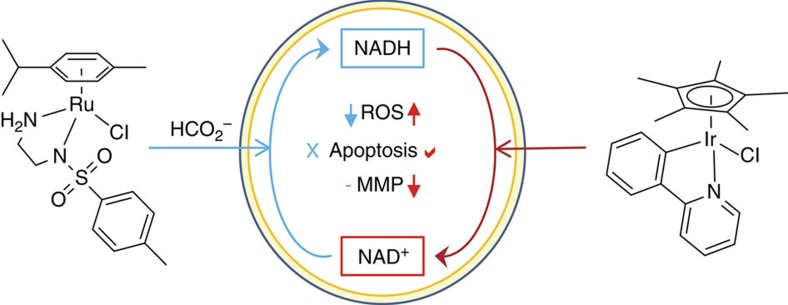
The contrasting mechanisms of action of two classes of organometallic anticancer catalysts. Illustrated is the induction of reductive stress in cancer cells by the Ru^II^ arene complexes reported here, and oxidative stress by Ir^III^ cyclopentadienyl complexes^8^. Both processes are mediated by changes in the NAD^+^/NADH redox couple. Ru^II^/formate donates hyride to NAD^+^, lowers the level of reactive oxygen species (ROS), but does not trigger changes in mitochondrial membrane potential (MMP) nor apoptosis. In contrast, the Ir^III^ complex accepts hydride from NADH, causes apoptosis, alters the MMP and generates oxidative stress via ROS production.

Whether such a combination of a catalyst and hydride transfer agent (formate) could be useful in therapy remains to be further investigated, but the importance of this work is more fundamental. There now exist organometallic catalysts that can perturb the NAD^+^/NADH ratio in cells in either direction: towards NADH using the Ru^II^ arene sulfonamido ethyleneamine/formate system reported here, or towards NAD^+^ using Ir^III^ cyclopentadienyl complexes (Fig. 5)^8^. Organometallic complexes are unique in their ability to achieve such redox modulation in living cells.

## Methods

### Transfer hydrogenation of NAD^+^

Complexes **1**–**7** were dissolved in MeOH*-d*_*4*_/D_2_O (5:1 *v*/*v*, 1.4 mM, 4 ml). Solutions of sodium formate (35 mM, 4 ml) and NAD^+^ in D_2_O (2.8 mM, 2 ml) were also prepared and incubated at 310 K. In a typical experiment, 200-μl aliquots of each solution were added to a 5-mm NMR tube and the pH* adjusted to 7.2±0.1 (total volume 0.635 ml, final concentrations: Ru complex 0.44 mM; NAD^+^ 0.88 mM; formate 11.02 mM; molar ratio 1:2:25). ^1^H NMR spectra were recorded at 310 K every 162 s until the completion of the reaction.

Molar ratios of NAD^+^ and NADH were determined by integrating the peaks at 9.33 and 6.96 p.p.m. (NAD^+^ and 1,4-NADH, respectively). The turnover number for the reaction was calculated as follows:


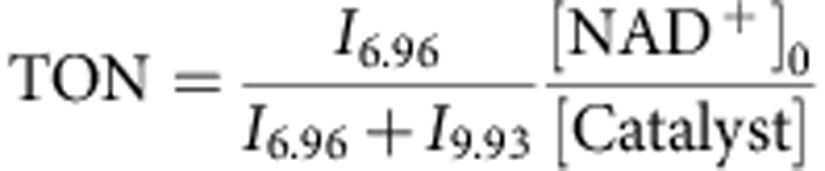


where *I*_*n*_ is the integral of the signal at *n* p.p.m. and [NAD^+^]_0_ is the concentration of NAD^+^ at the start of the reaction.

### Cell culture

A2780 human ovarian carcinoma and MRC5 human fetal lung fibroblasts were obtained from the European Collection of Cell Cultures. Both cell lines were grown in Roswell Park Memorial Institute medium (RPMI-1640) supplemented with 10% of fetal calf serum, 1% of 2 mM glutamine and 1% penicillin/streptomycin. All cells were grown as adherent monolayers at 310 K in a 5% CO_2_-humidified atmosphere and passaged at ca. 70–80% confluency.

### *In vitro* growth inhibition assays

The antiproliferative activity of complexes **1**–**8** was determined in A2780 ovarian cancer cells and MRC5 fibroblasts. Briefly, 96-well plates were used to seed 5,000 cells per well. The plates were left to pre-incubate with drug-free medium at 310 K for 48 h before adding different concentrations of the compounds to be tested. A drug exposure period of 24 h was allowed. After this, supernatants were removed by suction and each well was washed with PBS. A further 72 h were allowed for the cells to recover in drug-free medium at 310 K. The sulforhodamine B (SRB) assay was used to determine cell viability. IC_50_ values, as the concentration that causes 50% cell death, were determined as duplicates of triplicates in two independent sets of experiments and their s.d. were calculated.

### Co-administration of Ru complexes with formate or acetate

Cell viability assays were carried out with complexes **1**–**8** in A2780 ovarian cancer cells. These experiments were carried out as described above with the following modifications: a fixed concentration of each Ru complex equal to 1/3 × IC_50_ was used in co-administration with three different concentrations of sodium formate or sodium acetate (0.5, 1.0 and 2.0 mM). To prepare the stock solution of the drug, the complex was dissolved in 5% dimethylsulfoxide and diluted in a 1:1 mixture of 0.9% saline:cell culture medium. This stock was further diluted using RPMI-1640 until working concentrations were achieved. Separately, stock solutions of sodium formate or sodium acetate were prepared in saline. The complex and formate were added to each well independently, but within 5 min of each other.

### IC_50_ of complex 2 on co-administration with formate

The antiproliferative activity towards A2780 ovarian cancer cells of complex **2** when co-administered with 2 mM sodium formate was determined. These experiments were performed as described above, using a fixed concentration of formate and variable concentrations of the Ru complex. The SRB assay was used to determine cell viability. IC_50_ values were determined as duplicates of triplicates in two independent sets of experiments and their s.d. were calculated.

### Ruthenium accumulation in cancer cells

Briefly, 1.5 × 10^6^ cells per well were seeded on a six-well plate. After 24 h of pre-incubation, the complexes were added to give final concentrations equal to IC_50_/3 and a further 24 h of drug exposure was allowed. After this time, cells were washed, treated with trypsin-EDTA, counted and cell pellets were collected. Each pellet was digested overnight in concentrated nitric acid (73%) at 353 K; the resulting solutions were diluted using double-distilled water to give a final concentration of 5% HNO_3_ and the amount of Ru taken up by the cells was determined by ICP–MS. These experiments did not include any cell recovery time in drug-free media; they were all carried out as duplicates of triplicates and the s.d. were calculated. This experiment was also carried out using complex **2** and co-administering 2 mM sodium formate.

### Ruthenium distribution in cancer cells

Cell pellets were obtained as described above, and were fractionated using the Fraction PREP kit from BioVision according to the supplier’s instructions. Each sample was digested overnight in concentrated nitric acid (73%) and the amount of Ru taken up by the cells was determined by ICP–MS. These experiments were all carried out in triplicate and the s.d. were calculated.

### NAD^+^/NADH determination

Experiments to determine the NAD^+^/NADH ratio in A2780 ovarian cancer cells exposed to complex **2** were carried out using the NAD^+^/NADH assay kit from Abcam (ab65348) according to the manufacturer’s instructions. Briefly, A2780 cells were seeded in six-well plates at a density of 1.0 × 10^6^ cells per well. After 24 h of pre-incubation, cells were treated with a fixed concentration of **2** equal to its IC_50_ in co-administration with three different concentrations of sodium formate (0, 0.5, 1 and 2 mM). These concentrations of sodium formate were also used on their own as a second set of negative controls. Drug exposure was allowed for 24 h, after which all supernatants were removed by suction and wells were washed with PBS. Cells were treated with trypsin, detached and counted before being pelleted by centrifugation. Cell pellets were extracted using the NAD/NADH extraction buffer and filtered using a 10-kDa-molecular weight cutoff filter. Samples were split into two, the first half was used to determine total NADt (NAD+NADH), and the second half to determine NADH after heating the samples at 333 K for 30 min. The absorbance at 450 nm was normalized against the protein content in each sample determined using the Bradford assay.

### Time dependence of NAD^+^/NADH ratio

Experiments to determine the time dependence of the NAD/NADH ratio in A2780 ovarian cancer cells exposed to complex **2** were carried out using the NAD^+^/NADH assay kit as described above with the following modifications. The drug exposure time was variable and included time points at 0, 2, 4, 12, 18 and 24 h; the concentration of complex **2** was fixed at IC_50_ levels, and the concentration of formate was 2 mM.

### ROS determination

Flow cytometry analysis of total induction of ROS in A2780 cells caused by exposure to complex **2** and sodium formate was carried out using the Total ROS detection kit (Enzo Life Sciences) according to the supplier’s instructions. Briefly, 1.0 × 10^6^ A2780 cells per well were seeded in a six-well plate. Cells were pre-incubated in drug-free medium at 310 K for 24 h in a 5% CO_2_-humidified atmosphere, after which they were exposed to either sodium formate (2 mM), complex **2** (concentration equal to IC_50_) or a combination of both. After 24 h of drug exposure, supernatants were removed by suction and cells were washed and harvested. Staining was achieved by resuspending the cell pellets in buffer containing the green fluorescent reagent. Cells were analysed in a Becton Dickinson FACScan Flow Cytometer using Ex/Em: 490/525 nm for the oxidative stress detection. Data were processed using Flowjo software. The experiment included cells treated with pyocyanin for 30 min as positive control.

### Mitochondrial membrane assay

Analysis of the changes of mitochondrial potential in A2780 cells after exposure to complex **2** and sodium formate was carried out using the Abcam, JC-10 Mitochondrial Membrane Potential Assay kit according to the manufacturer’s instructions. Briefly, 1.0 × 10^6^ cells were seeded in six-well plates and left to incubate for 24 h in drug-free medium at 310 K in a humidified atmosphere. Drug solutions were added (sodium formate 2 mM, complex **2** at a concentration equal to IC_50_ or a combination of both, in triplicate experiments) and the cells were left to incubate for a further 24 h under similar conditions. Supernatants were removed by suction and each well was washed with PBS before detaching the cells using trypsin-EDTA. Staining of the samples was done in flow cytometry tubes protected from light, incubating for 30 min at room temperature. Samples were immediately analysed on a Beckton Dickinson FAC Scan with fluorescence detection. Data were processed using FlowJo software. This experiment included carbonyl cyanide *m*-chlorophenyl hydrazine as positive control.

### Induction of apoptosis

Flow cytometry analysis of apoptosis in A2780 cells caused by exposure to complex **2** and sodium formate were carried out using the Annexin V-FITC Apoptosis Detection Kit (Sigma Aldrich) according to the manufacturer’s instructions. Briefly, A2780 cells were seeded in six-well plates (1.0 × 10^6^ cells per well), pre-incubated for 24 h in drug-free media at 310 K, after which they were exposed to either sodium formate (2 mM), complex **2** (concentration equal IC_50_) or a combination of both. Cells were harvested using trypsin and stained using PI/Annexin V-FITC. After staining, cell pellets were analysed in a Becton Dickinson FACScan Flow Cytometer. For positive-apoptosis controls, A2780 cells were exposed for 2 h to staurosporine (1 μg ml^−1^). Cells for apoptosis studies were used with no previous fixing procedure as to avoid nonspecific binding of the annexin V-FITC conjugate.

### Induction of necrosis

Flow cytometry analysis of necrosis in A2780 cells caused by exposure to complex **2** and sodium formate was carried out using a Apotosis/Necrosis detection kit (Abcam) according to the manufacturer’s instructions. Briefly, A2780 cells were seeded in six-well plates (1.0 × 10^6^ cells per well), pre-incubated for 24 h in drug-free media at 310 K, after which they were exposed to either sodium formate (2 mM), complex **2** (concentration equal IC_50_) or a combination of both. Cells were harvested using trypsin and stained using the membrane-impermeable nuclear dye 7-ADD (Ex/Em 546/647 nm). After staining in the dark, cell pellets were analysed in a Becton Dickinson FACScan Flow Cytometer. For positive-necrosis controls, A2780 cells were exposed to methanol.

### Cellular membrane integrity

Flow cytometry analysis of cellular membrane integrity of A2780 cells caused by exposure to complex **2** and sodium formate were carried out using the CytoPainter assay (Abcam) according to the manufacturer’s instructions. Briefly, A2780 cells were seeded in six-well plates (1.0 × 10^6^ cells per well), pre-incubated for 24 h in drug-free media at 310 K, after which they were exposed to either sodium formate (2 mM), complex **2** (concentration equal IC_50_) or a combination of both. Cells were harvested using trypsin and stained in the dark using CytoPainter Red (Ex/Em 583/603 nm), which reacts with cell surface amines in compromised membranes. After staining, cell pellets were analysed in a Becton Dickinson FACScan Flow Cytometer.

## Author contributions

J.J.S.-B., I.R.-C., A.H. and P.J.S. designed the research. J.J.S.-B., I.R.-C. and A.H. performed research. J.J.S.-B., I.R.-C., A.H. and P.J.S. analysed data and J.J.S.-B., I.R.-C. and P.J.S. wrote the paper.

## Additional information

**How to cite this article:** Soldevila-Barreda, J. J. *et al*. Transfer hydrogenation catalysis in cells as a new approach to anticancer drug design. *Nat. Commun*. 6:6582 doi: 10.1038/ncomms7582 (2015).

## Supplementary Material

Supplementary Information Supplementary Figures 1-5, Supplementary Tables 1-11, Supplementary Methods and Supplementary References

## Figures and Tables

**Figure 1:**
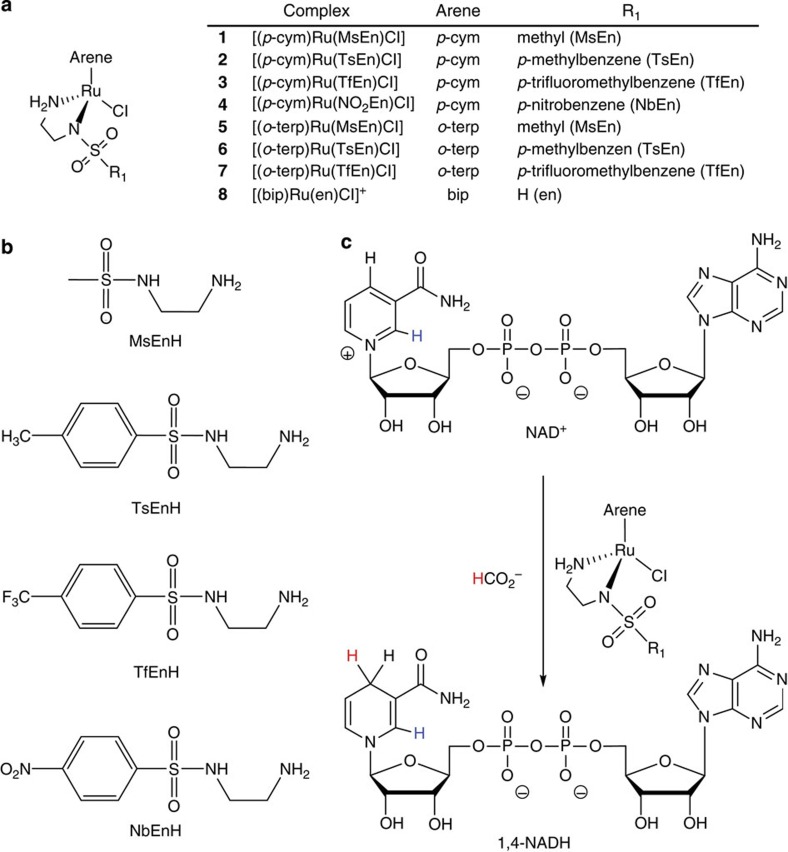
Complexes studied in this work and the catalytic reaction. (**a**) Structure of Ru complexes, (**b**) structures of the ligands and (**c**) catalytic conversion of NAD^+^ into 1,4-NADH mediated by formate.

**Table 1:** pK_a_* values for aqua adducts of catalysts 1–4 (and 8 for comparison) and turnover frequencies for transfer hydrogenation of NAD^+^ using formate.

Complexes		pK_a_*	TOF (h^−1^) D_2_O/MeOH*-d*_*4*_#	TOF (h^−1^) D_2_O
**1**	[(*p*-cym)Ru(MsEn)Cl]	9.86±0.01	1.25±0.03	1.11±0.02
**2**	[(*p*-cym)Ru(TsEn)Cl]	9.78±0.06	2.88±0.06	1.58±0.04
**3**	[(*p*-cym)Ru(TfEn)Cl]	9.71±0.01	5.7±0.3	3.06±0.05
**4**	[(p-cym)Ru(NO_2_En)Cl]	9.56±0.04	9.6±0.3	4.1±0.1
**8**	[(bip)Ru(en)Cl]^+^	7.71±0.01†	—	—

pK_a_*, pKa value determined in deuterated solvent; TOF, turnover frequency.

^#^23% MeOH*-d*_*4*_/77% D_2_O.

^†^ ref. 31, pK_a_ value (pK_a_* 7.85).
