# Supplementary material for: Transfer hydrogenation catalysis in cells as a new approach to anticancer drug design
Source: Nat Commun. 2015 Mar 20;6:6582. doi: 10.1038/ncomms7582 (PMC4383003; doi:10.1038/ncomms7582)
Supplement: Supplementary Information — Supplementary Figures 1-5, Supplementary Tables 1-11, Supplementary Methods and Supplementary References [file ncomms7582-s1.pdf]

1. Supplementary Figures.

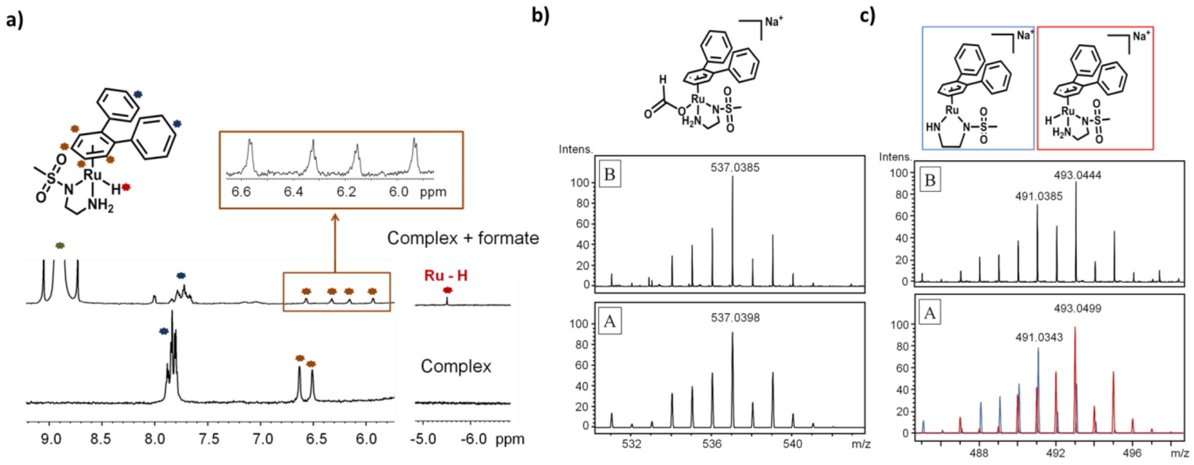

Supplementary Figure 1: Kinetics of transfer hydrogenation reactions with NAD$^+$: **a)** ^{1}H NMR spectra of the reaction between complex **5** and sodium formate (ratio 1:1000). The experiment was performed in D_2O, pH* 7.2 and temperature 310 K. **Orange** bound arene, **green** formate, **blue** phenyl groups of the arene, and **red** hydride. **b)** ^{1}H NMR spectra for the reaction mixture of complex **5** and formate. (A) Calculated spectra of hydride adduct of complex **5** (red)**,** complex **5** – HCl (blue), (B) experimental spectrum. **c)** High resolution mass spectrum for the reaction mixture of complex **5** and formate. (A) Calculated spectrum for the formate adduct of complex **5,** (B) experimental spectrum.

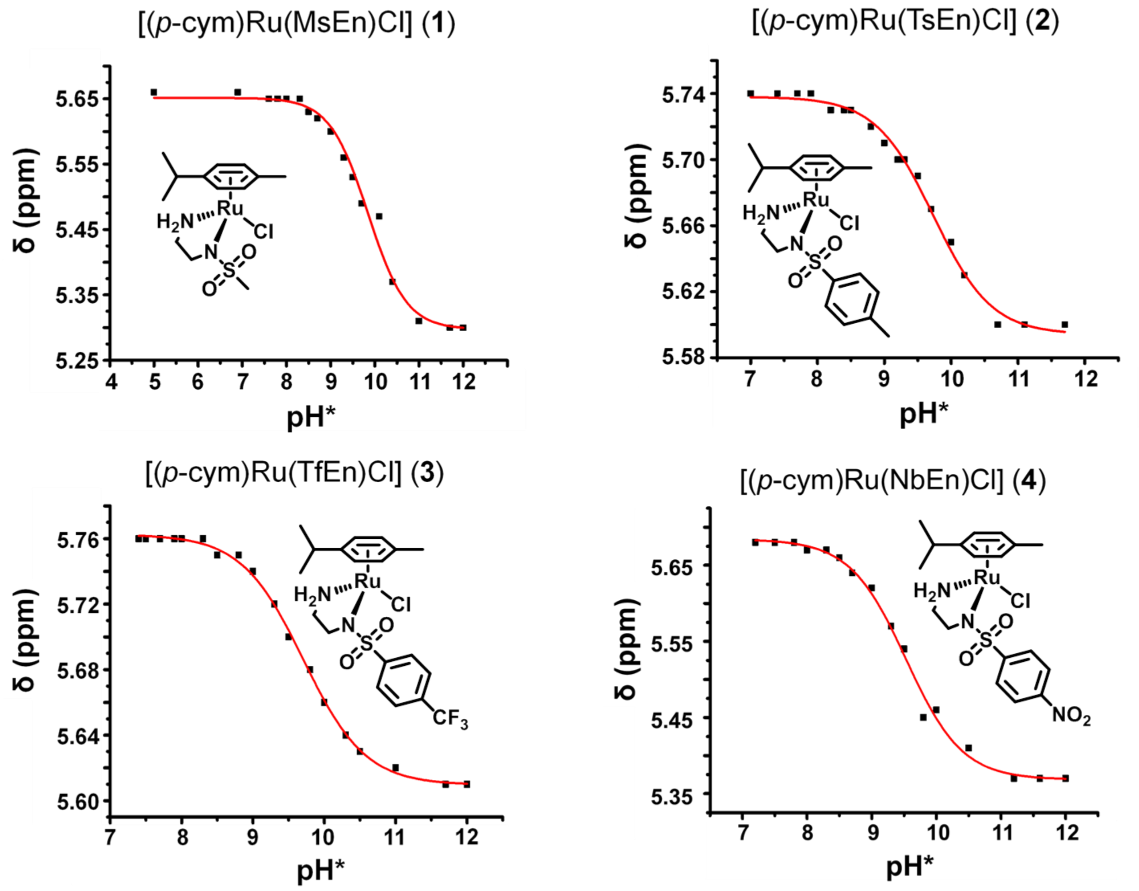

Supplementary Figure 2: ^{1}H NMR pH* titration curves for the aqua adducts of complexes **1-4** (for pK_a* values see Table 1).

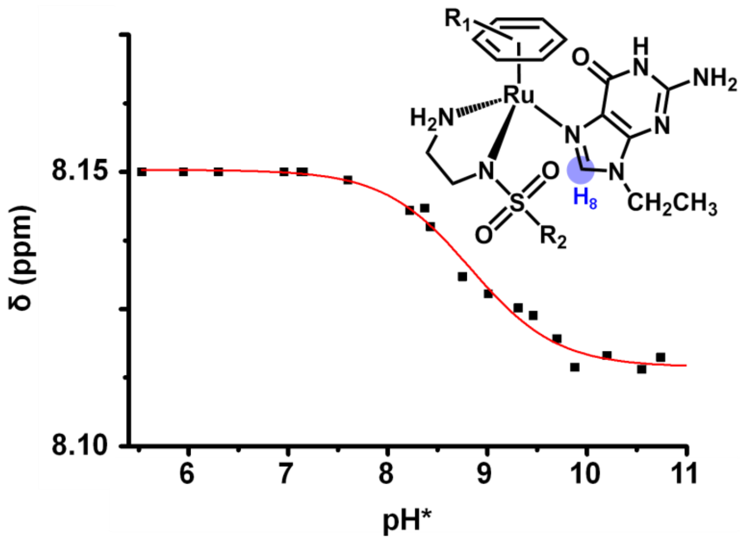

Supplementary Figure 3: Dependence of the H_8 ^{1}H NMR chemical shift of [(*p*-cym)Ru(TsEn)(9-EtG)]$^+$ on pH*. A pK_a* 8.78 ± 0.04 was determined using the Henderson-Hasselbalch equation.

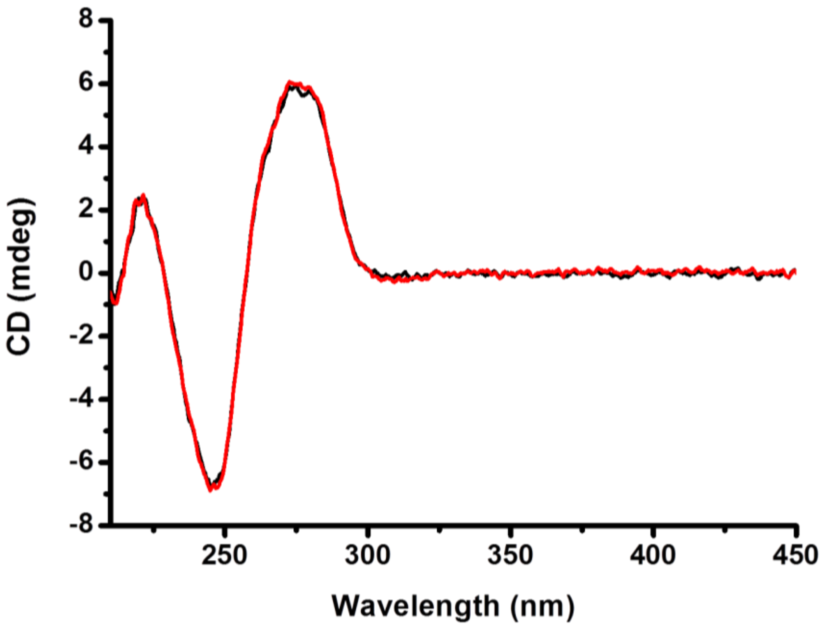

Supplementary Figure 4: Circular dichroism spectra of CT-DNA (1 mM) in red and CT-DNA (1 mM)/complex **2** (mol ratio 3:1) in black. Spectra were recorded under the same conditions, using (A) cacodylate buffer (2 mM, pH 7.4) and NaCl (20 mM) at 298 K, (B) PBS buffer (2 mM, pH 7.4) and NaCl (20 mM).

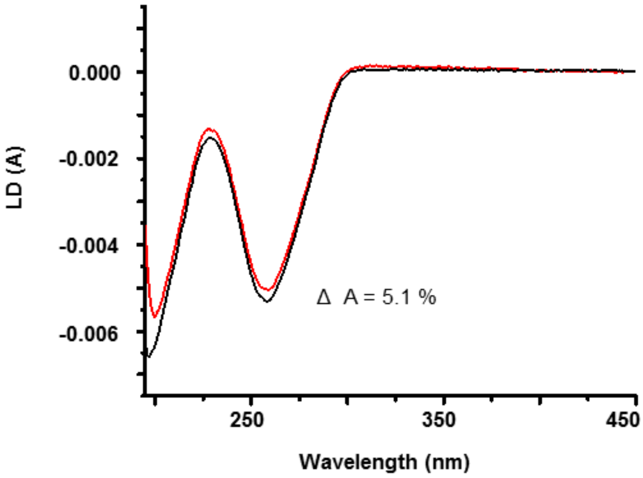

Supplementary Figure 5: LD spectra of solutions containing CT-DNA (black) and CT-DNA and complex **2** [(*p*-cym)Ru(TsEn)Cl] (mol. ratio 1:1, red) in 2 mM cacodylate buffer (pH 7.4) and 20 mM NaCl.

2. Supplementary tables

Supplementary Table 1: Retention times for complexes 1-7 reverse phase HPLC, where chelating ligand X-SO_2-En = NbEn, TfEn, TsEn or MsEn (for structures see Fig. 1).

Complex		Retention time (min)
[(*p*-cym)Ru(MsEn)Cl]	(**1**)	9.5
[(*p*-cym)Ru(TsEn)Cl]	(**2**)	15.36
[(*p*-cym)Ru(TfEn)Cl]	(**3**)	18.78
[(*p*-cym)Ru(NbEn)Cl]	(**4**)	16.13
[(*o*-terp)Ru(MsEn)Cl]	(**5**)	15.2
[(*o*-terp)Ru(TsEn)Cl]	(**6**)	19.77
[(*o*-terp)Ru(TfEn)Cl]	(**7**)	22.19

Supplementary Table 2: Relevant mass-to-charge ratios of products from the reaction between sodium formate and complex **5** at pH 7.3 ± 0.1 detected in the high resolution-mass spectrum

Proposed chemical formulae		Calculated (m/z)	Found (m/z)
$\mathbf{C_{21}H_{23}N_2O_2RuS^+}$	$[M\text{-}Cl]^+$	469.0518	469.0533
$\mathbf{C_{21}H_{22}N_2NaO_2RuS^+}$	$[M\text{-}HCl+Na]^+$	491.0343	491.0377
$\mathbf{C_{21}H_{24}N_2NaO_2RuS^+}$	$[M(H)+Na]^+$	493.0499	493.0456
$\mathbf{C_{22}H_{24}N_2NaO_4RuS^+}$	$[M(HCOO)+Na]^+$	537.0392	537.0396

Supplementary Table 3: Antiproliferative activity of complexes **1-8** in A2780 ovarian cancer cells and their cellular accumulation (expressed as ng Ru per million cells). The latter experiments were carried out using equipotent concentrations of the complexes (1/3 x IC_{50}) and 24 h of drug exposure, without recovery time. All the experiments were performed as duplicates of triplicates in independent experiments and the error bars were calculated as the standard deviation from the mean.

	Complex	IC_{50} (μM)	Cell Ru (ng x 10^6 cells)
**1**	[(*p*-cym)Ru(MsEn)Cl]	11.9 ± 0.8	0.16 ± 0.02
**2**	[(*p*-cym)Ru(TsEn)Cl]	13.6 ± 0.6	0.15 ± 0.04
**3**	[(*p*-cym)Ru(TfEn)Cl]	14.2 ± 0.8	0.31 ± 0.04
**4**	[(*p*-cym)Ru(NO_2En)Cl]	14.7 ± 0.6	n/d
**5**	[(*o*-terp)Ru(MsEn)Cl]	12.4 ± 0.9	2.8 ± 0.3
**6**	[(*o*-terp)Ru(TsEn)Cl]	15.8 ± 0.8	2.1 ± 0.4
**7**	[(*o*-terp)Ru(TfEn)Cl]	21.2 ± 0.1	2.4 ± 0.3
**8**	[(bip)Ru(en)Cl]$^+$	2.17 ± 0.08	n/d

Supplementary Table 4: Binding constants for formation of 9-EtG adducts of complexes **1-4** determined by ^{1}H NMR. The experiments were performed in D_2O, pH* 7.2 ± 0.1 and 310 K. Equilibrium was reached by the time the first ^{1}H NMR spectrum was recorded (< 10 min).

**Complex**		**Binding constant (mM^{-1})**
[(*p*-cym)Ru(MsEn)Cl]	**(1)**	105.4 ± 2.3
[(*p*-cym)Ru(TsEn)Cl]	**(2)**	104.9 ± 3.9
[(*p*-cym)Ru(TfEn)Cl]	**(3)**	60.5 ± 6.0
[(p-cym)Ru(NbEn)Cl]	**(4)**	92.8 ± 1.0
$[(bip)Ru(en)Cl]^+$	**(8)**	60.2[a]

[a] Reference [2]

Supplementary Table 5: Mass-to-charge (m/z) ratios for the 9-EtG adducts of complexes **1-5**.

Complex		Chemical Formula	Calc. m/z	Found m/z
[(*p*-cym)Ru(MsEn)(9-EtG)]$^+$	**(1-G**)	$C_{20}H_{32}N_7O_3RuS^+$	552.1330	552.1337
[(*p*-cym)Ru(TsEn)(9-EtG)]$^+$	(**2-G**)	$C_{26}H_{36}N_7O_3RuS^+$	628.1644	628.1647
[(*p*-cym)Ru(TfEn)(9-EtG)]$^+$	(**3-G**)	$C_{26}H_{33}F_3N_7O_3RuS^+$	682.1362	682.1362
[(*p*-cym)Ru(NbEn)(9-EtG)]$^+$	(**4-G**)	$C_{25}H_{33}N_8O_5RuS^+$	659.1339	659.1338

Supplementary Table 6: Melting temperature (T_m) of CT-DNA in the presence of **8** or complex **2** (ratio 1:3, complex: base pairs-DNA). The reactions were carried out in cacodylate buffer (2 mM, pH 7.4), and samples incubated for 2 h at 310 K.

**Complex**	**[NaCl]**	**T_m (K)**	**ΔTm[a]**
**-**	20 mM	332.3 ± 1.0	-
**8**	20 mM	340.2 ± 0.6	7.9**
**2**	20 mM	327.5 ± 0.7	-4.9**
**-**	2 mM	322.7 ± 1.3	-
**8**	2 mM	336.9 ± 0.8	14.0**
**2**	2 mM	320.3 ± 1.6	-2.4

[a] variation in melting temperature between free DNA and DNA in the presence of the corresponding complex. * $p < 0.5$ from free DNA using a T-test analysis. ** $p < 0.01$ from free DNA using a T-test analysis

Supplementary Table 7: Percent cell viability of A2780 ovarian cancer cells exposed to equipotent concentrations of ruthenium complexes **1-3** and **5-8** (1/3 x IC_{50}) for 24 h and different concentrations of sodium formate (0, 0.5, 1 and 2 mM). The experiments included 48 h of pre-incubation and 72 h of drug recovery, both in drug-free medium at 310 K in a 5% CO_2 humidified atmosphere. All the experiments were performed as duplicates of triplicates in independent experiments and the error bars were calculated as the standard deviation from the mean.

		Cell viability (%)			
		Formate (mM)			
	Complex	0	0.5	1	2
**1**	[(*p*-cym)Ru(MsEn)Cl]	70 ± 1	61± 1	40.0 ± 0.4	1.5 ± 0.1
**2**	[(*p*-cym)Ru(TsEn)Cl]	68 ± 1	49.7 ± 0.8	38.2 ± 0.6	1.5 ± 0.1
**3**	[(*p*-cym)Ru(TfEn)Cl]	71 ± 2	60± 2	39.4 ± 2.0	19 ± 2
**5**	[(*o*-terp)Ru(MsEn)Cl]	73.59 ± 0.04	71.9 ± 0.4	44.2 ± 0.2	5.8 ± 0.6
**6**	[(*o*-terp)Ru(TsEn)Cl]	72.8 ± 0.9	59 ± 3	43.6 ± 1.4	22.2 ± 0.8
**7**	[(*o*-terp)Ru(TfEn)Cl]	40 ± 2	20.8 ± 0.6	11.9 ± 0.6	2.5 ± 0.7
**8**	$[(bip)Ru(en)Cl]^+$	70 ± 2	71 ± 1	61 ± 1	39 ± 1

Supplementary Table 8: Antiproliferative activity of complex **2** in A2780 ovarian cancer cells when co-administered with different concentrations of sodium formate (0, 0.5, 1 and 2 mM) and amount of Ru accumulated by the cells. All the experiments were performed as duplicates of triplicates in independent experiments and the error bars were calculated as the standard deviation from the mean.

	Formate concentration (mM)			
	0	0.5	1	2
IC_{50} (μM)	13.6 ± 0.6	10.8 ± 0.6	5.4 ± 0.8	1.0 ± 0.2
Cell Ru (ng x 10^6 cells)	0.15 ± 0.03	0.145 ± 0.009	0.129 ± 0.005	0.13 ± 0.02

Supplementary Table 9: Cellular distribution of complex **2** (as % Ru) in A2780 ovarian cancer cells when co-administered with different concentrations of sodium formate (0 and 2 mM). All the experiments were performed as duplicates of triplicates in independent experiments and the error bars were calculated as the standard deviation from the mean.

Formate	%Ru<br>Cytosol	Membrane	Cytoskeleton	Nuclei
0 mM	50.5 ± 2.4	41.6 ± 1.4	1.9 ± 0.5	6.0 ± 2.8
2 mM	52.0 ± 3.0	35.7 ± 7.2	1.0 ± 0.8	11.3 ± 4.5

Supplementary Table 10: Antiproliferative activity of complex **2** in A2780 ovarian cancer cells and normal MRC5 human fetal lung fibroblasts, when co-administered with different concentrations of sodium formate (0 and 2 mM). All the experiments were performed as duplicates of triplicates in independent experiments and the error bars were calculated as the standard deviation from the mean.

	Formate concentration (mM)			
	0	0.5	1	2
A2780, IC_{50} (μM)	13.6 ± 0.6	10.8 ± 0.6	5.4 ± 0.8	1.0 ± 0.2
MRC5, IC_{50} (μM)	48.5 ± 0.4	-	-	5.2 ± 0.8

Supplementary Table 11: Cell viability (%) of A2780 ovarian cancer cells exposed for 24 h to equipotent concentrations of complexes **2** and **8** (1/3 x IC_{50}) co-administered with different concentrations of sodium acetate (0, 0.5, 1 and 2 mM). The experiments involved 48 h of pre-incubation and 72 h of drug recovery, both in drug-free medium at 310 K in a 5% CO_2 humidified atmosphere. All the experiments were performed as duplicates of triplicates in independent experiments and the error bars were calculated as the standard deviation from the mean.

		Cell viability percentages (%)			
Complex		Acetate concentration (mM)			
		0	0.5	1	2
**2**	[(*p*-cym)Ru(TsEn)Cl]	70.8 ± 0.6	69.4 ± 0.9	68.9 ± 0.3	67.1 ± 0.6
**8**	$[(bip)Ru(en)Cl]^+$	75.4 ± 2	73.1 ± 0.8	72 ± 1	71 ± 1

3. Supplementary methods

3.1. Synthesis of the complexes

[(*p*-cym)Ru(MsEn)Cl] (1). [(*p*-cym)$RuCl_2$]$_2$ (84.2 mg, 0.14 mmol) and MsEnH (101.6 mg, 0.35 mmol) were placed in a round-bottom flask to which 2-propanol (50 mL) and triethylamine (146 μL, 1.05 mmol) were added. The solution was heated under reflux in a nitrogen atmosphere overnight, after which the solvent was removed on a rotary evaporator to give a light brown powder. The crude product was redissolved in dichloromethane and washed with brine, after which the organic layer was dried over $MgSO_4$ and filtered. The brown-reddish powder obtained after removal of the solvent *in vacuo* was recrystallized from methanol/ether (1:10 v/v) by standing in a freezer for two months at 253 K.

Yield: 45.3 mg, 40.4 %.

^{1}H NMR (400 MHz, methanol-*d$_4$*): δ_H 5.59 (s, 2H), 5.42 (d, 2H, J_{HH} = 5.7 Hz), 2.85 (sept, 1H, J_{HH} = 7.1 Hz), 2.74 (s, 3H), 2.134 (s, 3H), 1.265 (d, 6H, J_{HH} = 7.1 Hz).

Elemental analysis. Found (calculated for $C_{13}H_{23}ClN_2O_2RuS$): C: 38.27 (38.28), H: 5.65 (5.68), N: 6.82 (6.87)

ESI-MS for $C_{13}H_{23}ClN_2O_2RuS$: $(M-Cl)^+$ 373.0 m/z.

[(*p*-cym)Ru(TsEn)Cl] (2). Complex **2** was obtained following the method described above for complex **1** using the ligand TsEnH (100.6 mg, 0.42 mmol). Recrystallization from methanol resulted in dark red crystals.

Yield: 58.8 mg, 36.7 %.

^{1}H NMR (400 MHz, acetone-*d$_6$*): δ_H 7.73 (d, 2H, J_{HH} = 8.3 Hz), 7.11 (d, 2H, J_{HH} = 8.3 Hz), 5.85 (s, 1H), 5.71 (d, 1H, J_{HH} = 5.6 Hz), 5.64 (d, 1H, J_{HH} = 5.6 Hz), 5.52 (d, 1H, J_{HH} = 5.6 Hz), 5.45 (d, 1H, J_{HH} = 5.6 Hz), 3.24 (s, 1H), 2.94 (sept, 1H, J_{HH} = 7.1 Hz), 2.84 (m, 1H), 2.68 (m, 1H), 2.31(s, 3H), 2.18(m, 1H), 2.15 (s, 3H), 1.27 (d, 3H, J_{HH} = 7.0 Hz), 1.24 (d, 3H, J_{HH} = 7.0 Hz).

Elemental analysis. Found (calculated for $C_{19}H_{27}ClN_2O_2RuS$)%: C 46.70 (47.15), H 5.62 (5.62), N 5.79 (5.79).

ESI-MS for $C_{19}H_{27}ClN_2O_2RuS$: $(M\text{-}Cl)^+$ 449.0 m/z.

[(*p*-cym)Ru(TfEn)Cl] (3). Complex **3** was obtained following the method described above for complex **1** using ligand TfEnH (125.0 mg, 0.47 mmol). Recrystallization was not successful and the product was used as isolated.

Yield: 171.1 mg, 85.3 %

^{1}H NMR (400 MHz, acetone-*d$_6$*): δ_H 8.00 (d, 2H, J_{HH} = 8.1 Hz), 7.62 (d, 2H, J_{HH} = 8.1 Hz), 5.74 (m, 1H), 5.51 (m, 2H), 5.41 (d, 1H), 4.23 (s, 1H), 3.27 (s, 1H), 3.10 (s, 1H), 2.76 (sept, 1H, J_{HH} = 6.9), 2.75 (m, 1H), 2.29 (m, 2H), 2.17 (s, 3H), 1.27 (m, 6H).

Elemental analysis. Found (calculated for $C_{19}H_{24}ClF_3N_2O_2RuS$) %: C 41.94 (42.42), H 4.45 (4.50), N 5.03 (5.21).

ESI-MS for $C_{19}H_{24}ClF_3N_2O_2RuS$: $(M\text{-}Cl)^+$ 503.0 m/z.

[(*p*-cym)Ru(NbEn)Cl] (4). Complex **4** was obtained following the method described above for complex **1** using ligand NbEnH (89.0 mg, 0.363 mmol). The crude product was recrystallized from methanol to give an orange crystalline powder.

Yield: 117.3 mg, 58.59 %.

^{1}H NMR (400 MHz, acetone-*d*$_6$): δH 8.25 (d, 2H, J_{HH} = 8.8 Hz), 8.02 (d, 2H, J_{HH} = 8.8 Hz), 5.81 (d, 1H, J_{HH} = 6.0 Hz), 5.65 (d, 1H, J_{HH} = 6.0 Hz), 5.56 ((d, 1H, J_{HH} = 6.0 Hz), 5.51 (d, 1H, J_{HH} = 6.0 Hz), 5.34 (s, 1H), 3.03 (m, 2H), 2.94 (septet, 1H, J_{HH} = 7.0 Hz), 2.76 (m, 1H), 1.35 (m, 6H, J_{HH1} = 7.0 Hz, J_{HH2} = 4.6 Hz).

Elemental analisys found (calculated for $C_{18}H_{24}ClN_3O_4RuS$) %: C 41.94 (41.98), H 4.64 (4.70), N 8.22 (8.16).

ESI-MS for $C_{18}H_{24}ClN_3O_4RuS$: $(M-Cl)^+$ 480.0 m/z.

[(*o*-terp)Ru(MsEn)Cl] (5). [(*o*-terp)$RuCl_2$]$_2$ (110.0 mg, 0.14 mmol) and MsEnH (79.3 mg, 0.27 mmol) were placed in a round-bottom flask and dissolved in 2-propanol (50 mL). Triethylamine (42 µL, 0.30 mmol) was added and the solution was heated overnight at reflux temperature under a nitrogen atmosphere, after which solvent was removed on a rotary evaporator to give a dark brown product. The crude product was redissolved in dichloromethane and washed with brine; the organic layer was dried over $MgSO_4$ and filtered. The solution was concentrated *in vacuo* and the product recrystallized from methanol to afford a brown solid.

Yield: 33.3 mg, 24.2 %.

^{1}H NMR (250 MHz, acetone-*d*$_6$): δH 7.57 (m, 4H), 7.39 (m, 6H), 6.23 (t, 1H, J_{HH} = 5.6 Hz), 6.05 (t, 1H, J_{HH} = 5.6 Hz), 6.02 (t, 1H, J_{HH} = 5.6 Hz), 5.80 (t, 1H, J_{HH} = 5.6 Hz),

5.10 (s, 1H), 3.36 (s, 1H), 2.94 (m, 1H), 2.78 (s, 3H), 2.75 (m, 1H), 2.31 (m,1H), 2.14 (m, 3H).

Elemental analysis. Found (calculated for $C_{21}H_{23}ClN_2O_2RuS$) %: C 49.15 (50.04), H 4.48 (4.60), N 5.56 (5.56).

ESI-MS for $C_{21}H_{23}ClN_2O_2RuS$: $(M\text{-}Cl)^+$ 469.0 m/z.

[(*o*-terp)Ru(TsEn)Cl] (6). Complex **6** was obtained using the ligand TsEnH (106.3 mg, 0.50 mmol) following the method described above for complex **5**. The crude product was recrystallized from methanol to give a brown powder.

Yield: 51.2 mg, 23.8 %.

^{1}H NMR (250 MHz, acetone-*d*$_6$): δH 7.76 (d, 2H, J_{HH} = 7.6 Hz), 7.22-7.60 (m, 12H), 6.33 (t, 1H, J_{HH} = 5.6 Hz), 6.08 (d, 1H, J_{HH} = 5.9 Hz), 6.05 (t, 1H, J_{HH} = 5.9 Hz), 5.85 (t, 1H, J_{HH} = 5.5 Hz), 5.15 (s, 1H), 3.31 (s, 1H), 2.98 (m, 1H), 2.70 (s, 3H), 2.47 (s, 3H).

Elemental analysis. Found (calculated for $C_{27}H_{27}ClN_2O_2RuS$) %: C 55.41 (55.90), H 4.77 (4.69), N 4.52 (4.83).

ESI-MS for $C_{27}H_{27}ClN_2O_2RuS$: $(M\text{-}Cl)^+$ 545.0 m/z.

[(*o*-terp)Ru(TfEn)Cl] (7). Complex **7** was obtained using the ligand TfEnH (100.2 mg, 0.37 mmol) following the method described above for complex **5**. The crude product was recrystallized from methanol to give a reddish-brown crystalline powder.

Yield: 58.8 mg, 24.9 %.

^{1}H NMR (250 MHz, acetone-*d*$_6$): δH 7.97 (d, 2H, J_{HH} = 8.4 Hz), 7.70 (d, 2H, J_{HH} = 8.4 Hz), 7.27-7.57 (m, 10H), 6.32 (t, 1H, J_{HH} = 5.7 Hz), 6.14 (t, 1H, J_{HH} = 5.7 Hz), 6.04 (t,

1H, J_{HH} = 5.7 Hz), 5.88 (t, 1H, J_{HH} = 5.7 Hz), 5.22 (s, 1H), 3.28 (s, 1H), 3.08 (m, 1H), 2.75 (m, 1H), 2.33 (m,1H), 2.14 (m, 1H),

Elemental analysis. Found (calculated for $C_{27}H_{24}ClF_3N_2O_2RuS$) %: C: 50.85 (51.14), H: 3.77 (3.82), N: 4.38 (4.42).

ESI-MS for $C_{27}H_{24}ClF_3N_2O_2RuS$: $(M-Cl)^+$ found 599.0 m/z.

3.2. Characterization of formate and hydride adducts.

Solutions of complexes **1-3** (1.4 mM in D_2O) and **5-7** (1.4 mM in MeOD-*d₄*/D_2O 2:9 v/v) were added to an aqueous solution of sodium formate (140 mM), pH* adjusted to 7.4 ± 0.1 and ^{1}H NMR spectra recorded for a period of 4 h. No signals corresponding to an hydride adduct were detected for complexes **1-3** and **7** in the high field region. However, Ru-H peaks assignable to the hydride adducts of complexes **5** and **6** were detected at -5.5 ppm and -5.6 ppm, respectively.

The HR-MS spectrum of the reaction mixture containing complex **5** and sodium formate in a mol ratio 1:1000 showed a peak at 493.04 m/z which was identified as the hydride adduct of the complex plus sodium ($C_{21}H_{24}N_2NaO_2RuS^+$). Interestingly, a peak at 537.04 m/z was also identified suggesting the existence of a stable formate adduct (Supplementary Table 2, Supplementary Figure 1). Formation of stable formate adducts and other carboxylate adducts has been previously reported for related complexes.[1] The lifetimes of both species are short, and none of the adducts could be isolated.

3.3. Aquation studies

Solutions of complexes **1-7** (1.4 mM, 10% MeOD-d_4/90% D_2O) were prepared and monitored by ^{1}H NMR spectroscopy. ^{1}H NMR spectra were recorded at 298 K over a

period of 24 h on a Bruker AV III 600 spectrometer (^{1}H = 600 MHz) using 5 mm diameter NMR tubes. All data processing was carried out using Topspin 2.1.

Aqua adducts of complexes **1-7** were prepared by treatment of a solution of the corresponding chlorido complex (10% MeOD-d_4/90% D_2O) with silver nitrate (1 mol equiv) overnight and followed by filtration through Celite to remove the AgCl formed.

3.4. pK_a* determination of Ru aqua complexes

Changes in the chemical shifts of the arene protons of the aqua adducts for complexes **1-4** with pH* over a range from 2 to 12 were followed by ^{1}H NMR spectroscopy. pH* values were measured at ambient temperature using a minilab IQ125 pH meter, pH sensor and referenced to KCl gel. The pH* was adjusted with KOD or $DClO_4$ solutions in D_2O. ^{1}H-NMR spectra were recorded at 298 K on a Bruker AV III 600 spectrometer (^{1}H = 600 MHz) using 5 mm diameter tubes. The data were fitted to the Henderson–Hasselbalch equation using Origin 7.5. (Supplementary Figure 2)

3.5. Hydrophobicity

The relative hydrophobicities of the Ru(II) complexes were determined from their retention times on reverse-phase HPLC, using a HP 1200 Series HPLC System (Agilent), and Agilent ZORBAX Eclipse Plus C-18 (250 x 4.6 mm, 5 μm pore size) column, eluted with solvents A (0.1% TFA/water) and B (0.1% TFA/acetonitrile): t = 0 – 10 % B, t = 25 – 70 % B, t = 30 – 70 % B, t = 31 – 10 % B and t = 36 – 10 % B over a 36 min. The sample injection volume was 50 μL, and flow rate 1 mL min^{-1}. The wavelength of detection was 254 nm with the reference wavelength 360 nm. Samples were prepared in double distilled water (ddw) or a mixture methanol (5%, HPLC grade)/ddw.

The HPLC retention times are listed in Supplementary Table 1. The hydrophobicity is dependent on the η^6-arene in the order *o*-terp > *p*-cym. The chelating ligand also greatly affects the hydrophobicity of the complexes, the more hydrophilic complexes being those containing MsEn and in the order MsEn < TsEn < NbEn < TfEn.

3.6. DNA experiments

DNA is believed to be the main target for $[(bip)Ru(en)Cl]^+$ (**8**). Due to the structural similarity of the complexes **1-5** with complex **8**, their binding to DNA was investigated.

Interaction with nucleobases

Initially, model nucleobases, 9-ethylguanine and 9-methyladenine were reacted with complexes **1-4** in D_2O, 310 K and pH* 7.2 ± 0.1. The binding of the complexes to 9-EtG or 9-MeA was monitored by ^1H-NMR spectroscopy. Peaks for the 9-EtG adducts were observed by the time the first spectrum was recorded (< 5 min after mixing), and no change in the ^1H NMR spectrum after 24 h was observed, indicating that the equilibrium was reached rapidly. The binding constants for formation of 9-EtG adducts determined by NMR were between 60 and 106 mM^{-1} (Supplementary Table 4). These values are in the same range as that from the ethylenediamine complex $[(bip)Ru(en)Cl]^+$ ($K_{9\text{-}EtG}$= 60.3 mM^{-1}),[2] which also binds strongly to DNA.[3, 4] The high resolution mass spectrum confirmed the formation of $[(\eta^6\text{-arene})Ru(XEn)(9\text{-}EtG)]^+$ (Supplementary Table 5). The interaction of complexes **1-4** with 9-MeA was also investigated, but no reaction was detected by ^1H-NMR.

It is well established that N7 of guanine is the preferred nucleotide binding site for many transition metals ions. [2-8] Furthermore, strong and selective binding to N7 of 9-EtG, has been observed for $[(\eta^6\text{-arene})Ru(en)Cl]^+$.[3, 4] A pH* titration of

$[(p\text{-cym})Ru(TsEn)(9\text{-EtG})]^+$ was used to confirm that the complex binds to N7. A plot of the chemical shift of H_8 from bound 9-EtG (Supplementary Figure 3), versus pH* gave a pK_a* of 8.77, which can be attributed to the protonation/deprotonation of N1 from coordinated 9-EtG and therefore we conclude that the binding of complex **2** to 9-EtG occurs though N7. [9]

Interaction with calf thymus DNA

Complex **2** was chosen as representative of the series. A stock solution of complex **2** (0.7 mM in 5 % acetonitrile/water) was prepared and aliquots of 66 µL were added to a solution of CT-DNA (final ratio 1:3 complex: base pairs-DNA) in water, 2 mM cacodylate buffer and 2 mM NaCl. The reaction mixture was incubated at 310 K, and aliquots of 300 µL were taken at different intervals during a period of 27 h. The DNA was then precipitated by addition of ethanol (700 µL, final concentration 70% v/v). Ru complexes which are strongly bound to CT-DNA will precipitate together with the DNA upon addition of ethanol, and, therefore the ruthenium concentration in the supernatant should decrease. However, no changes in the Ru concentration were detected by ICP-MS showing that the interaction between the complex **2** and DNA is weak (t = 0 h / 64.2 ± 0.7 µM, t = 24 h/ 65.1 ± 0 .3 µM free Ru). In contrast complex **8** was found to bind strongly to DNA (t = 0 h / 58.3 ± 0.3 µM, t = 24 h / 48.5 ± 0.6 µM), as reported previously. [10]

Thermal stability of CT-DNA

The melting temperature of CT-DNA in the presence and absence of complex **2** [(*p*-cym)Ru(TsEn)Cl] was determined by UV-Vis spectroscopy (Supplementary Table 6). Higher melting temperatures indicate increased stabilization of the DNA, which is the effect produced by DNA-intercalators and often by direct metal coordination to

the nucleobases.[11] Destabilization of DNA can result in a decrease of the melting temperature.

In the presence of complex **8**, the melting temperature of CT-DNA increased In agreement with the literature, [12] by 14 K, in 2 mM NaCl, 2 mM cacodylate buffer. This increase is due to Ru binding to DNA as well as intercalation. [12] However, upon increasing the chloride concentration by *ca*. 10x (20 mM NaCl) the melting temperature of CT-DNA in the presence of complex **8**, increased only by 7 K. This effect has previously been reported and it is attributed to the partial inhibition of hydrolysis, and, therefore, inhibition of coordination to DNA. [12, 13]

Experiments performed in the presence of complex **2,** in 2 mM cacodylate buffer and 2 mM NaCl, showed a 4 K decrease in the melting temperature. However, in 20 mM NaCl, no decrease of the melting temperature was observed. These experiments therefore again suggest that complex **2** interacts only weakly with DNA.

Conformational changes of CT-DNA

Interaction of a metallodrug with DNA may induce changes in DNA conformation. Furthermore, strong binding to DNA can induce chirality in the metal complex, generating a CD signal. [14] CD spectra of CT-DNA in the presence of different concentrations of complex **2** were recorded (Supplementary Figure 4). No changes in the CD spectra were observed.

Flow linear dichroism can be used to study the structure and relative orientation of molecules. The technique has been used to study interactions between DNA and ligands or metal complexes. [14]

In a typical spectrum of CT-DNA, negative bands at 260 and 190 nm are observed. These bands correspond to the absorbance of DNA bases which are oriented with an angle of 80° from the DNA backbone. [14] A series of LD experiments was performed using complex **2**. Only a slight decrease in the intensity of the LD signals was observed suggesting that the complex has only weak affinity for DNA.

3.7. Inductively coupled plasma mass spectrometry (ICP-MS)

Ruthenium (^{101}Ru) content was determined using ICP-MS Agilent technologies 7500 series. Data acquisition was carried out on ICP-MS Top (B.03.05) and analysed using Offline Data Analysis (B.03.05). Ru Standard solution (ruthenium chloride 1000 ppm in 10 % v/v hydrochloric acid) was purchased from Inorganic Ventures. Calibration curves were prepared using double deionised water (ddw) with 3% nitric acid, range between 100 and 1 ppb (9 points). Samples were freshly prepared in ddw with 3% metal free nitric acid. Readings were made in no-gas mode.

4. Supplementary references:

1. Betanzos-Lara, S. *et al.* Organometallic Ruthenium and Iridium Transfer-Hydrogenation Catalysts Using Coenzyme NADH as a Cofactor *Angew. Chem. Int. Ed.* **51**, 3897-3900 (2012).
2. Peacock, A.F.A. *et al.* Chloro Half-Sandwich Osmium(II) Complexes: Influence of Chelated N,N-Ligands on Hydrolysis, Guanine Binding, and Cytotoxicity. *Inorg. Chem.* **46**, 4049-4059 (2007).
3. Chen, H., Parkinson, J.A., Morris, R.E. & Sadler, P.J. Highly Selective Binding of Organometallic Ruthenium Ethylenediamine Complexes to Nucleic Acids : Novel Recognition Mechanisms. *J. Am. Chem. Soc.*, 173-186 (2003).
4. Chen, H. *et al.* Organometallic Ruthenium(II) Diamine Anticancer Complexes: Arene-Nucleobase Stacking and Stereospecific Hydrogen-Bonding in Guanine Adducts. *J. Am. Chem. Soc.* **124**, 3064-3082 (2002).
5. van Rijt, S.H., Peacock, A.F.A., Johnstone, R.D.L., Parsons, S. & Sadler, P.J. Organometallic osmium(II) arene anticancer complexes containing picolinate derivatives. *Inorg. Chem.* **48**, 1753-1762 (2009).
6. Peacock, A.F.A. *et al.* Tuning the reactivity of osmium (II) and ruthenium (II) arene complexes under physiological conditions. *J. Am. Chem. Soc.* **128**, 1739-1748 (2006).
7. Melchart, M., Habtemariam, A., Parsons, S., Moggach, S.A. & Sadler, P.J. Ruthenium (II) arene complexes containing four- and five-membered monoanionic O , O-chelate rings. *Inorg. Chim. Acta.* **359**, 3020-3028 (2006).
8. Liu, H.-K. *et al.* Diversity in Guanine-Selective DNA Binding Modes for an Organometallic Ruthenium Arene Complex. *Angew. Chem. Int. Ed.* **45**, 8153-8156 (2006).
9. Kampf, G., Kapinos, L.E., Griesser, R. & Sigel, H. Comparison of the acid–base properties of purine derivatives in aqueous solution. Determination of intrinsic proton affinities of various basic sites †. *J. Chem.Soc., Perkin Trans.* **2**, 1320-1327 (2002).
10. Liu, H.-K. & Sadler, P.J. Metal Complexes as DNA Intercalators. *Acc. Chem. Res.* **44**, 349-359 (2011).
11. Kelly, J.M., Tossi, A.B., McConnell, D.J. & OhUigin, C. A study of the interactions of some polypyridylruthenium(II) complexes with DNA using fluorescence spectroscopy, topoisomerisation and thermal denaturation. *Nucleic Acids Res.* **13**, 6017-6034 (1985).
12. Novakova, O. *et al.* DNA Interactions of Monofunctional Organometallic Ruthenium (II) Antitumor Complexes in Cell-free Media †. *Biochemistry* **42**, 11544-11554 (2003).
13. Pizarro, A., Habtemariam, A. & Sadler, P. Activation Mechanisms for Organometallic Anticancer Complexes, in *Medicinal Organometallic Chemistry*. (eds. G. Jaouen & N. Metzler-Nolte) 21-56 (Springer Berlin Heidelberg, 2010).
14. Dafforn, T., Norden, B. & Rodger, A. *Linear Dichroism and Circular Dichroism*, Vol. 331, Edn. 1st edition. (The Royal Society of Chemistry, Cambridge, UK; 2011).
